# Evaluation of the TMJ by means of Clinical TMD Examination and MRI Diagnostics in Patients with Rheumatoid Arthritis

**DOI:** 10.1155/2014/328560

**Published:** 2014-08-26

**Authors:** Silke Witulski, Thomas J. Vogl, Stefan Rehart, Peter Ottl

**Affiliations:** ^1^Private Practice, Hauptstraße 5-7, 63808 Haibach, Germany; ^2^Center for Radiology, Institute of Diagnostic and Interventional Radiology, Johann Wolfgang Goethe University, Theodor-Stern-Kai 7, 60590 Frankfurt, Germany; ^3^Clinic for Orthopedics and Trauma Surgery, Agaplesion Markus Hospital, Wilhelm-Epstein-Straße 4, 60431 Frankfurt, Germany; ^4^Department of Prosthodontics and Materials Science, University of Rostock, Strempelstraße 13, 18057 Rostock, Germany

## Abstract

This study included 30 patients with diagnosed rheumatoid arthritis (RA) and 30 test subjects without RA (control group). The objective of the study was to examine both groups for the presence of temporomandibular disorders (TMD) and morphological changes of the temporomandibular joint (TMJ). All individuals were examined using a systematic detailed clinical TMD examination as well as magnetic resonance imaging (MRI). The clinical TMD examination yielded significant differences between the RA patients and the control group concerning crepitus of the TMJ, and palpation tenderness of the masticatory muscles as well as the unassisted mandibular opening. The evaluation of the MRI images for the RA group showed significantly more frequent deformations of the condyle, osteophyte formations and erosions in the condylar compacta, and degenerative changes in the spongiosa. Increased intra-articular accumulation of synovial liquid and signs of inflammatory changes of the spongiosa were only found in the RA group. Statistical analysis showed a significant correlation between crepitus and specific osteoarthrotic changes (MRI), respectively, and between crepitus and a complete anterior disk displacement without reduction (MRI). The duration of the RA disease correlated neither with the anamnestic and clinical dysfunction index by Helkimo nor with RA-specific MRI findings.

## 1. Introduction

Rheumatoid arthritis (RA) as a chronic progressive autoimmune disease causes progressive joint changes that are irreversible. Involvement of the temporomandibular joint (TMJ) is stated in the literature as varying greatly from 2 to 88% [1–4]. This is due on one hand to the very different selection of the patient populations and to age distribution and the duration or the severity of the RA, respectively. On the other hand, the study criteria and methods differ with regard to collecting the anamnestic data, the definition of diagnostic criteria, and the imaging techniques used. However, the majority of studies showed that approximately 50% of RA patients develop a clinical involvement of the TMJ [5–9].

Frequent symptoms in case of TMJ involvement are pain accompanying movements of the mandible, restricted movements of the mandible, and joint sounds as well as radiating head and facial pain. Often, patients do not realize the association between these complaints and the RA [3, 10].

Clinical TMD examination is of decisive importance for the diagnosis of temporomandibular disorders (TMD) and especially for the evaluation of the temporomandibular joint (TMJ). In complex cases it is necessary to include imaging procedures to support and confirm the diagnosis. Among the various imaging procedures, the formerly popular X-ray techniques have lost much of their importance due to the insufficient or nonexistent representation of the soft tissues in and around the TMJ and the resulting limited validity of the resulting images. Compared to X-ray techniques, magnetic resonance imaging (MRI) of the TMJ results in no radiation load and facilitates representation of the articular hard-tissue and soft-tissue situation.

While MRI is accepted as the “gold standard” in imaging procedures for diagnosis of the TMJ relevant studies on the validity of using MRI for evaluating the TMJ in RA patients do not exist.

The objective of this study was to compare between patients suffering from RA and healthy test subjects (control group) for the presence of TMD and morphological changes of the TMJ by using a systematic detailed clinical TMD examination and a systematic method for evaluating MRI images. In addition the correlation between clinical findings and MRI findings should be evaluated.

## 2. Patients and Methodology

### 2.1. RA Group (Patients with RA)

In collaboration with the Department of Orthopedics and Trauma Surgery of Agaplesion Markus Hospital, 30 consecutive patients (27 females/3 males) over the age of 18 (inclusion criterion) with an already diagnosed RA as primary disease were summoned for clinical examination at the Department of Prosthodontics, School of Dentistry, Johann Wolfgang Goethe University, Frankfurt, Germany. The diagnosis of the RA was secured by the ACR/EULAR Rheumatoid Arthritis Classification Criteria [11] as well as the course of the illness in combination with the typical clinical characteristics and chemical findings. Existing infectious diseases and drug abuse were exclusion criteria. The study design presented no selection with regard to existence of temporomandibular disorders.

The age of the RA patients ranged from 28 to 76 years. The mean age was 56.9 ± 10.4 years. The duration of RA disease averaged 12.8 ± 12.4 years. The patients were told not to take any of their rheumatologist-prescribed, take-when-needed nonsteroidal antirheumatic drugs (NSADs) and analgesics on examination day in order not to influence findings of the clinical TMD examination based on the presence of tenderness. Taking other medications (e.g., cortisone, methotrexate, and leflunomide) was not interdicted; an interruption was not justifiable, given that their therapeutic effects depend on a specific ongoing dosage.

Authorization by the Ethical Review Committee of the Johann Wolfgang Goethe University, Frankfurt, Germany, was obtained (reference number 122/99). The examinations were conducted after the patients were informed and had provided their written consent.

### 2.2. Group N (Normal Population)

Thirty test subjects (15 females/15 males) who did not reveal a history in accordance with RA nor did they present typical clinical characteristics of RA served as control group. Their ages ranged from 18 to 69 years. The average age was 31.5 ± 11.6 years.

### 2.3. Clinical TMD Examination

Using a systematic detailed evaluation form, an experienced dentist collected history data and performed the clinical TMD examination. The following key parameters were evaluated.

- *Palpation (Bilateral).* Check of pain on palpation of the TMJs (lateral pole/posterior attachment) and muscles.
- *TMJ Sounds.* Auscultation of the TMJs during opening and closing of the mandible for clicking or crepitus (initial, intermediate, and terminal).
- *Range of Motion of Mandible.* Measurement of the maximum unassisted mandibular opening as well as right lateral excursion, left lateral excursion, and protrusion.

The results were summarized and classified using the anamnestic and clinical dysfunction indices developed by Helkimo [12]. The anamnestic dysfunction index classifies history data on three levels: Ai0 (subjectively symptom-free), AiI (mild symptoms), and AiII (severe symptoms). The clinical dysfunction index allows classification of the functional state of the craniomandibular system based on five groups of symptoms that are assessed during the clinical TMD examination. These result in the dysfunction groups Di0 (clinically symptom-free), DiI (mild symptoms), DiII (moderate symptoms), and DiIII (severe symptoms).

### 2.4. Additional Clinical Examination (Orthopaedic Tests)

Additional findings for the clinical TMD examination were collected by means of several orthopaedic tests [13].

- *Static Pain Tests.* These tests serve for the clinical evaluation of the masticatory muscles during mandibular opening and closing, during right lateral and left lateral excursion. The muscles that coordinate the respective movement were evaluated for pain.
- *Compression Tests.* Using five separate tests, different compressive loads (toward cranial, toward dorsocranial, and toward dorsal) were applied to the TMJ.
- *Traction/Translation Tests.* Using four separate tests, various tensile loads (toward caudal, toward ventrocaudal, toward medial, and toward lateral) were applied to the TMJ.

### 2.5. MRI Examination and Evaluation

The MRI images of the TMJ were produced at the Center for Radiology, Institute of Diagnostic and Interventional Radiology of Johann Wolfgang Goethe University at Frankfurt, Germany. The magnetic resonance images were taken using a magnetic resonance tomograph (1.5 Tesla) (MAGNETOM Symphony; Siemens, Erlangen, Germany) in combination with bilateral TMJ surface coils (double ring array coil; Siemens, Erlangen, Germany) featuring SE (spin echo) sequences (T1-weighted, proton density- (PD-) weighted, or T2-weighted; 5 to 7 slices; 3 mm slice thickness). The imaging was done without using contrast media.

The evaluation of the MRI images was done by means of a systematic detailed and validated evaluation form developed by Ottl et al. [14, 15]. For right (R) and left (L) TMJs the condylar morphology (macromorphology, compacta, and spongiosa), the disk morphology, and the fossa morphology as well as the tubercular morphology, the condyle/fossa relationship, and the disk position on two planes (both with closed mouth and open mouth) as well as the signal intensity (condyle, joint space/bilaminar zone (PD- or T2-weighted)) were evaluated (see Supplementary Figures 1(a)-1(b) in Supplementary Material available online at http://dx.doi.org/10.1155/2014/328560).

### 2.6. Statistical Analysis

All data were stored and analyzed using the SPSS Statistical Package 20.0 (SPSS Inc., Chicago, Illinois, USA). Descriptive statistics were computed for continuous and categorical variables. The statistics computed included median and interquartile range of continuous variables and frequencies and relative frequencies of categorical factors. Testing for differences of continuous variables between the study groups was accomplished by the 2-sample *t*-test for independent samples (parametric test, normally distributed data) or the Mann-Whitney *U* test (nonparametric test), as appropriate. Test selection was based on evaluating the variables for normal distribution employing the Kolmogorov-Smirnov test. Comparisons between the study groups for categorical variables were done using the chi-square test or Fisher's exact test.

To show whether and how strongly pairs of variables are related correlations were assessed using Spearman's rho correlation coefficient.

All *P* values resulted from two-sided statistical tests and values of *P* < 0.05 were considered to be statistically significant.

## 3. Results

### 3.1. Clinical TMD Examination and Orthopaedic Tests

**Figure 1:**
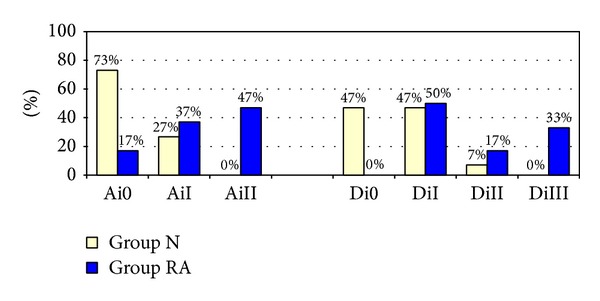
Percentage distribution of anamnestic (Ai) and clinical dysfunction indices (Di) by Helkimo in test subjects of the control group (group N, *n* = 30 subjects) and the group of patients with rheumatoid arthritis (group RA, *n* = 30 patients). Ai0 (subjectively symptom-free), AiI (mild symptoms), AiII (severe symptoms), Di0 (clinically symptom-free), DiI (mild symptoms), DiII (moderate symptoms), and DiIII (severe symptoms).

The anamnestic data of all individuals yielded a more frequent presentation of subjective symptoms and dysfunctions from the sample of RA patients (Group RA) than from the control group (group N) (Table 1). The anamnestic dysfunction index developed by Helkimo showed significantly higher values within the RA group (Mann-Whitney *U* test, *P* < 0.001) (Figure 1).

Tables 2 and 3 summarize the results of the clinical TMD examination. Palpation tenderness of the TMJs was determined in both groups; however, the percentage was higher in the RA group. Clicking of the TMJ was also evident in both groups. Crepitus was only present in the RA group (*P* < 0.001). The comparison of both groups with regard to palpation tenderness of the muscles also saw higher values in the RA group and yielded a statistically significant result (*P* < 0.001). Maximum unassisted mandibular opening was limited more frequently in the RA group (*P* = 0.021). Similarly, the results of the orthopaedic tests showed higher values concerning unilateral/bilateral pain for all evaluation parameters in group RA (Table 4). The classification of the results using the Helkimo clinical dysfunction index yielded more frequent presence of moderate to severe dysfunction within the RA group (Mann-Whitney *U* test, *P* < 0.001) (Figure 1). The *P* values for the statistical analysis between both groups for the individual parameters of the clinical TMD examination and orthopaedic tests can be found in Table 5.

### 3.2. MRI Evaluation

The results of the MRI evaluation are shown in Tables 6 and 7. Using the T1-weighted images, regular, convex* condylar morphology* was present in the majority of group N (73% right side/67% left side), while this was true for fewer patients in the RA group (50% right side/53% left side). Flattening was frequently seen in both groups (group N: 20% right side/33% left side; group RA: 30% right side/17% left side). Deformations were only recorded in group RA (40% right side/43% left side).

The* condylar compacta* showed erosions and osteophyte formations to a larger extent for the RA group (67% right side/80% left side) than for group N. The* condylar spongiosa* showed no degenerative changes in group N; however, it did so in half the patients in group RA.

The* fossa morphology* evaluation yielded similar results.* Disk morphology* in both groups showed a large variety of forms. It most frequently presented as a biconcave structure (group N: 53% right side/73% left side; group RA: 60% right side/57% left side). In group RA, additionally, an overall/central thinning of the disk morphology (37% right side/30% left side) was found as well as deformation, perforation, and a destroyed disk.

The* condyle/fossa relationship* (closed mouth/parasagittal) in group RA frequently revealed a posterior (37% right side/33% left side) and caudal orientation (47% right side/43% left side) of the condyle.

With regard to the* condyle/disk relationship* (closed mouth/parasagittal) in group RA a high percentage of partial anterior disk displacements (DD) with reduction (33% right side/37% left side) was detected as well as a complete anterior DD without reduction (17% right side/20% left side).

*Increased signal intensity* (intra-articular liquid accumulation) in the* bilaminar zone* or in the* joint space* occurred at a significantly higher frequency in group RA (80% right side/90% left side) (*P* = 0.001).* Increased signal intensity* of the* condylar spongiosa* was seen significantly more often in the left TMJ (*P* = 0.011) of the RA group (13% right side/23% left side). The *P* values for the statistical analysis of both groups for the individual MRI parameters can be seen in Tables 8 and 9.

A statistically significant correlation between the presence of crepitus and specific osteoarthrotic changes in the MRI findings (condyle deformation (R) *P* = 0.013/(L) *P* = 0.007; osteophyte formation/condyle (R) *P* = 0.013/(L) *P* = 0.074; erosions/fossa (R) *P* = 0.014/(L) *P* = 0.022) could be demonstrated as well as between a clinically restricted mandibular opening and a total anterior DD without reduction shown in the MRI findings ((R) *P* = 0.02/(L) *P* = 0.003).

The statistical analysis could not demonstrate a significant correlation between clinical palpation tenderness (posterior attachment), a pain induced by passive compression, or a pain induced by ventrocaudal translation, respectively, and the presence of increased signal intensity (intra-articular liquid accumulation) in the MRI findings. Furthermore, the duration of the RA disease does not correlate (Spearman's rho correlation coefficient) with the anamnestic and clinical dysfunction index by Helkimo (*r* < 0.2, *P* > 0.7) nor with selected MRI findings (deformation of the condyle, osteophyte formations/erosions of the condylar compacta, degenerative changes in condylar spongiosa, disk deformation, and complete anterior disk displacement without reduction).

## 4. Discussion

All questions during the interview with the investigated persons that relate to TMJ symptoms received positive answers more frequently in the RA group. Studies by Kallenberg et al. [10] and Helenius et al. [1, 16] also document severe symptoms of TMD in RA patients.

More frequent tenderness on palpation of the TMJ was observed in the RA group than in group N. Analysis of the literature shows that data concerning TMJ pain on palpation in RA patients are subject to a wide variety. Lin et al. [3] were able to establish this finding in 35.7% of patients and Holmlund et al. [17] in 86% of RA patients. Other studies demonstrated pain on TMJ palpation in approximately 50% of RA patients [7, 16].

TMJ sounds were present in both groups. However, crepitus only appeared in the RA group. Concerning this parameter the results in the literature vary as well, but there is consensus that crepitus is preponderantly observed in RA patients as opposed to healthy test subjects [3, 16–18].

In the present study, 93% of the individuals in the RA group demonstrated palpation tenderness of the muscles. Helenius et al. [16] were able to demonstrate a similarly high percentage, while other studies detected lower values [3, 7, 17]. Moreover, the evaluation of the static pain tests showed significant differences between the two groups. Muscle pain is an indicator of the extent of the dysfunction and the pathological changes in the masticatory system [16]. The evaluation of the unassisted mandibular opening also differs widely in the literature. Tegelberg and Kopp revealed a reduced mandibular opening in RA patients compared to healthy test subjects [18]. Other studies demonstrated no significant differences between the two groups [3, 5, 9, 19]. Larheim et al. discovered that motion as a condylar rotation can still take place despite complete destruction of the condyle and missing condylar translation [2]. In RA patients the maximum mandibular opening can therefore create a misleading impression of the TMJ's condition. In the present study, the values for maximum unassisted mandibular opening in the RA group were lower than in group N. But only in 13% of RA patients was the maximum unassisted mandibular opening less than 40 mm.

The classification of the present study's results using the anamnestic and clinical dysfunction index developed by Helkimo clearly shows the higher prevalence of TMD in the RA group compared to group N. Other authors were also able to demonstrate this fact [10, 20, 21].

The moderate correlation between the two dysfunction indices points out that the clinical findings do not necessarily match with the subjectively described symptoms of the RA patients. The anamnestic data showed that RA patients assess the importance of the TMD symptoms differently because of problems in their other joints. Several authors were also able to observe this aspect [3, 10, 19, 22]. The structural differences of the TMJ compared to other joints, especially the bilaminar zone, which provides efficient blood drainage for liquid accumulations, could possibly counter a swelling of the joint or joint pain [3]. The medications that were not interdicted, while the NSAD and analgesics were halted according to the study design of the present study, can also reduce inflammatory episodes like pain symptoms. In this regard detailed statements concerning the examined patients are often missing in the literature.

Similarly, there is no evidence of a typical temporal relationship between RA diagnoses and TMD manifesting [9, 10], which corresponds with a moderate correlation of the anamnestic and the clinical dysfunction index with the duration of the RA disease. Moen et al. were able to demonstrate a correlation between the clinical dysfunction index developed by Helkimo and the Disease Activity Score DAS 28 and thus RA's disease activity [23]. A comparison with the results of the compression/traction/translation tests is not possible because of missing studies in this regard. Contrary to the high frequency of positive findings to be expected, TMJ pain presented to a lesser degree in the RA group, which may possibly be influenced by the effect of the base medication.

Assessment of rheumatic changes by use of X-ray techniques has been applied in numerous studies on bony structures [5, 9, 24]. Condylar bone deformations and erosions are the most important signs of rheumatoid destruction; the joint space and the position of the condyle can also be evaluated by means of X-ray technology. Thanks to modern MRI technology, it has become possible to image the anatomical structures and especially the soft tissues of the TMJ in more detail. Typical MRI findings in the context of RA are bone deformations and destructions, osteophyte formations, and erosions as well as abnormal disk structures all the way to a disk being destroyed and the presence of intra-articular liquid accumulations as well as sclerosing and inflammatory processes inside the condylar spongiosa [1, 2, 25–27]. Comparing the studies demonstrates that the results with respect to the individual MRI parameters vary widely, which can be caused by differing study designs as well as different study populations. Similarly, a differentiated system for evaluating MRI images is lacking. Little attention has been paid to date to the differing morphologies of the disk, the condyle/fossa relationship, and the condyle/disk relationship. Systematic documentation of MRI findings of the TMJ using a detailed evaluation form [14, 15] streamlines the evaluation process. It facilitates a graded evaluation, ensuring consistent documentation of all main and subsidiary findings.

More than half the patients in the present study's RA group presented a biconcave or biplanar disk shape, however, combined with a large number of additional signs of degeneration, such as flattening or central, respectively, and overall thinning, which could stem from load stress as well as pathological processes. Disk deformation, disk perforation, and destruction of the disk were only present in the RA group. A few authors see in the disk morphology an indicator for the progression of the rheumatic involvement [26, 28]. A correlation between the duration of RA and a disk deformation could not be demonstrated statistically in the present study.

With respect to the condyle/disk relationship, it is noteworthy that there is less prevalence of a disk displacement among RA patients than among individuals who present with TMD [2, 26]. A disk displacement existing for a longer duration can induce degenerative changes in the corresponding joint surfaces [29–31]. Foucart et al. were able to demonstrate a high correlation between the presence of osteoarthrosis and an anterior disk displacement without reduction in TMD patients [32]. On the other hand, other authors interpret the disk displacement not as the cause of an osteoarthrosis but as its sequela [33]. Noteworthy in the present study is that the majority of patients presenting a total anterior disk displacement without reduction had a deformed disk as well as a deformed condyle. This might be interpreted as an indication to the previously described, reciprocally interacting pathological processes involving disk displacements and osteoarthrotic changes. Hence, degenerative changes play a dominant role in RA. Nevertheless, a condylar deformation as well as osteophyte formation can also be a progression from an internal derangement. If degenerative changes in the presence of a disk displacement dominate in patients with RA, it is difficult to classify those osteoarthrotic changes with regard to their development. They can be a consequence of an internal derangement as well as a sign of rheumatoid processes.

The RA patient group in this study is represented by 90% females. As the gender distribution of RA in the total population is 3 : 1 in favor of females the distribution within the patient group is in good agreement with these data.

In RA patients the inflammation-related processes within the synovia are responsible for development of an intra-articular accumulation of synovial liquid. In the literature the presence of a joint effusion is associated with arthrogenic pain as well as disk displacements, bone marrow edemas, and necroses, less so with osteoarthrotic changes [34–36]. Although more TMJ pain sensations were recorded for the RA patients percentage-wise in comparison with the control group in this study, it was, however, not to the extent that could be expected according to the manifestation of the joint effusion. The systematic, combined treatment using cytostatics like infliximab and methotrexate can reduce TMJ pain and stimulate an increase in anti-inflammatory cytokines and receptors in the synovial fluid [37]. This fact supplies a possible explanation for why a joint effusion in the context of rheumatoid arthritis does not necessarily have to manifest itself as pain.

## 5. Conclusions

There is a certain probability that a RA patient may develop signs and symptoms of TMD in the course of time. A timely TMD examination is considered necessary, since the present study shows no correlation between the duration of the RA disease and the dysfunction indices by Helkimo, and between the duration of the RA disease and the RA-specific MRI findings. When a RA is mentioned in patient history, a timely diagnosis based on clinical examination and MRI should be performed in order to recognize pathological conditions of the TMJ and to treat them appropriately. Revealing parameters and hence relevant indicators from clinical examination are crepitus, palpation of the muscles, and static pain tests. Relevant MRI findings are condylar deformation, osteophyte formation and erosions of the condylar compacta or of the fossa articularis, degenerative changes in the condylar spongiosa, and intra-articular liquid accumulations in the bilaminar zone or joint space.

## Supplementary Material

Figure 1(a): Evaluation form for MRI images of the temporomandibular joint (side 1). (condylar morphology (macromorphology, compacta, spongiosa), disk morphology, fossa morphology, tubercular morphology, condyle/fossa relationship).
Figure 1(b): Evaluation form for MRI images of the temporomandibular joint (side 2). (disk position on two planes (both with closed mouth and open mouth), signal intensity (condyle, joint space/bilaminar zone (PD- or T2-weighted)).

## Figures and Tables

**Table 1:** History data of test subjects in the control group (group N) and the group of patients with rheumatoid arthritis (group RA).

Anamnesis	Group N	Group RA
Sample size (*n* individuals)	30	30

Did you suffer an accident or a blow to the head/neck region?	33%	27%
Do/did you feel pain or discomfort in/on		
(i) Head (general)?	3%	40%
(ii) Temples?	0%	20%
(iii) Ears/TMJ region?	13%	30%
(iv) Neck?	20%	63%
(v) Shoulders?	7%	73%
(vi) Other joints?	7%	97%
Does/did your discomfort affect your sense of well-being or performance?	7%	83%
Are/were chewing, mouth opening, mouth closing, and/or other mandibular movements impaired or painful?	0%	70%
TMJ sounds (left/right)?	27%	40%

**Table 2:** Results of the clinical TMD examination in test subjects of the control group (group N) and the group of patients with rheumatoid arthritis (group RA).

Examination parameter TMJ palpation tenderness	Group N		Group RA	
Sample size (*n* individuals)	30		30	

TMJ (lateral pole) uni- or bilateral	10%		46%	
TMJ (post. attachment) uni- or bilateral	3%		20%	
	left	right	left	right
TMJ (lateral pole) mild pain	6%	10%	27%	10%
TMJ (lateral pole) pain	0%	0%	13%	6%
TMJ (post. attachment) mild pain	3%	3%	10%	10%
TMJ (post. attachment) pain	0%	0%	0%	0%

Examination parameter TMJ sounds	Group N		Group RA	

Sample size (*n* individuals)	30		30	

TMJ sounds uni- or bilateral	20%		33 %	
	left	right	left	right
TMJ clicking	20%	10%	13%	13%
TMJ crepitus	0%	0%	33%	30%

Examination parameter Muscle palpation tenderness	Group N		Group RA	

Sample size (*n* individuals)	30		30	

Muscles uni- or bilateral	37%		93%	
M. masseter pars superficialis	7%		33%	
M. masseter pars profundus	7%		63%	
M. temporalis pars anterior	3%		20%	
M. temporalis pars posterior	3%		7%	
Tendon of M. temporalis	7%		67%	
M. pterygoideus lateralis	0%		83%	
Posterior mandibular region	3%		67%	
Submandibular region	3%		10%	
Suboccipital/neck muscles	23%		57%	

**Table 3:** Results of the clinical TMD examination in test subjects of the control group (group N) and the group of patients with rheumatoid arthritis (group RA).

Examination parameter Range of motion of mandible (interquartile range)	Group N	Group RA
Sample size (*n* individuals)	30	30

Max. unassisted mandibular opening (mm)	54 (47–57)	51 (43–54.5)
Max. assisted mandibular opening (mm)	55 (49–61.5)	53 (46–58)
Max. right lateral excursion (mm)	9 (7.5–10)	9 (6.25–10)
Max. left lateral excursion (mm)	9 (8–10)	10 (9–12)
Max. protrusion (mm)	8 (7–8.8)	7 (5–8)

**Table 4:** Results of the orthopaedic tests in test subjects of the control group (group N) and the group of patients with rheumatoid arthritis (group RA).

Examination parameter Uni- or bilateral pain	Group N	Group RA
Sample size (*n* individuals)	30	30

Static pain test (mandibular opening)	7%	43%
Static pain test (mandibular closing)	7%	30%
Static pain test (right lateral excursion)	10%	20%
Static pain test (left lateral excursion)	3%	13%
Compression tests right	3%	10%
Compression tests left	3%	23%
Traction/translation tests right	10%	17%
Traction/translation tests left	3%	23%

**Table 5:** Statistical analysis of results from the clinical TMD examination and orthopaedic tests in test subjects of the control group (group N, *n* = 30 subjects) and the group of patients with rheumatoid arthritis (group RA, *n* = 30 patients).

Examination parameter	Statistical test	*P* values left	*P* values right
TMJ palpation (lateral pole)	Fisher's exact test	0.008	0.612
TMJ palpation (intra-articular)	Fisher's exact test	0.612	0.354
TMJ clicking	Fisher's exact test	0.731	1.0
TMJ crepitus	Fisher's exact test	0.001	0.002
Muscle palpation	Mann-Whitney *U* test	0.001	0.001
Static pain tests	Mann-Whitney *U* test	0.001	0.012
Compression tests	Mann-Whitney *U* test	0.027	0.321
Traction/translation tests	Mann-Whitney *U* test	0.024	0.453

**Table 6:** Results of the MRI evaluation for test subjects of the control group (group N, *n* = 30 subjects) and the group of patients with rheumatoid arthritis (group RA, *n* = 30 patients).

MRI findings	Group N		Group RA	
	left	right	left	right
Sample size (*n* individuals/*n* TMJs)	30	30	30	30

Condylar morphology				
Convex	67%	73%	53%	50%
Flattening	33%	20%	17%	30%
Deformation	0%	0%	43%	40%
Gable shaped/pointed angle	3%	7%	3%	3%
Compacta (condyle)				
No pathological findings	90%	78%	20%	23%
Erosion	0%	6%	40%	47%
Osteophyte formation	10%	16%	80%	67%
Spongiosa (condyle)				
No pathological findings	100%	100%	50%	47%
Degeneration	0%	0%	50%	53%
Fossa morphology				
No pathological findings	97%	90%	37%	43%
Erosion	0%	0%	17%	23%
Osteophyte formation	3%	10%	60%	57%
Disk morphology				
Biconcave	73%	53%	57%	60%
Overall/central thinning	3%	7%	30%	37%
Biplanar	23%	43%	17%	20%
Flattening in the marginal area	20%	27%	10%	13%
Thickening in the marginal area	7%	0%	0%	0%
Deformation	0%	0%	17%	13%
Perforation	0%	0%	3%	7%
Destroyed/fragmented	0%	0%	7%	3%
Overall thickening	3%	3%	3%	0%

**Table 7:** Results of the MRI evaluation for test subjects of the control group (group N, *n* = 30 subjects) and the group of patients with rheumatoid arthritis (group RA, *n* = 30 patients). (DD = disk displacement.)

MRI findings	Group N		Group RA	
	left	right	left	right
Sample size (*n* individuals/*n* TMJs)	30	30	30	30

Condyle/fossa relationship (closed mouth)				
Centered	90%	87%	60%	47%
Anterior oriented	3%	0%	7%	17%
Posterior oriented	7%	13%	33%	37%
Cranial oriented	3%	3%	23%	20%
Caudal oriented	17%	13%	43%	47%
Disk position parasagittal (closed mouth)				
Regular	83%	67%	27%	33%
Partial anterior DD with reduction	10%	30%	37%	33%
Partial anterior DD without reduction	0%	0%	3%	0%
Complete anterior DD with reduction	3%	0%	0%	0%
Complete anterior DD without reduction	3%	0%	20%	17%
No evaluation possible	0%	3%	13%	17%
Signal intensity PD/T2 (condyle)				
Increased signal intensity	0%	0%	23%	13%
Signal intensity PD/T2 (bilaminar zone/joint space)				
Increased signal intensity	7%	3%	90%	80%

**Table 8:** Statistical analysis (Fisher's exact test) of results from the MRI evaluation in test subjects of the control group (group N, *n* = 30 subjects) and the group of patients with rheumatoid arthritis (group RA, *n* = 30 patients).

Examination parameter	*P* values left TMJ	*P* values right TMJ
Condyle morphology		
Convex	0.430	0.110
Flattening	0.233	0.552
Deformation	0.001	0.001
Gable shaped/pointed angle	1.000	1.000
Compacta (condyle)		
No pathological findings	0.001	0.001
Erosion	0.001	0.001
Osteophyte formation	0.001	0.001
Spongiosa (condyle)		
No pathological findings	0.001	0.001
Degeneration	0.001	0.001
Fossa morphology		
No pathological findings	0.001	0.001
Erosion	0.024	0.011
Osteophyte formation	0.001	0.001
Disk morphology		
Biconcave	0.279	0.795
Overall/central thinning	0.012	0.010
Biplanar	0.748	0.095
Flattening in the marginal area	0.472	0.333
Deformation	0.052	0.112
Perforation	1.000	0.492
Destroyed/fragmented	0.492	1.000

**Table 9:** Statistical analysis (Fisher's exact test) of results from the MRI evaluation in test subjects of the control group (group N, *n* = 30 subjects) and the group of patients with rheumatoid arthritis (group RA, *n* = 30 patients).

Examination parameter	*P* values left TMJ	*P* values right TMJ
Condyle/fossa relationship (closed mouth)		
Centered	0.015	0.002
Anterior oriented	1.000	0.052
Posterior oriented	0.021	0.072
Cranial oriented	0.052	0.103
Caudal oriented	0.047	0.010
Disk position parasagittal (closed mouth)		
Regular	0.001	0.019
Partial anterior DD with reduction	0.030	1.000
Partial anterior DD without reduction	1.000	—
Complete anterior DD with reduction	1.000	—
Complete anterior DD without reduction	0.103	0.052
Signal intensity (PD/T2) (condyle)		
Increased signal intensity	0.011	0.112
Signal intensity PD/T2 (bilaminar zone/joint space)		
Increased signal intensity	0.001	0.001
