# Supplementary material for: Evaluation of the TMJ by means of Clinical TMD Examination and MRI Diagnostics in Patients with Rheumatoid Arthritis
Source: Biomed Res Int. 2014 Aug 26;2014:328560. doi: 10.1155/2014/328560 (PMC4160621; doi:10.1155/2014/328560)
Supplement: Supplementary file 1 — Figure 1(a): Evaluation form for MRI images of the temporomandibular joint (side 1). (condylar morphology (macromorphology, compacta, spongiosa), disk morphology, fossa morphology, tubercular morphology, condyle/fossa relationship). Figure 1(b): Evaluation form for MRI images of the temporomandibular joint (side 2). (disk position on two planes (both with closed mouth and open mouth), signal intensity (condyle, joint space/bilaminar zone (PD- or T2-weighted)). [file 328560.f1.pdf]

Universitätsmedizin Rostock

University of Rostock
Rostock/Germany
Department of Prosthodontics
and Materials Science

MRI TMJ Evaluation

Patient No.: _________ Family name: ___________ Given name: __________ Date of birth: ________
Referred by: _________________________ MRI examination by: _______________________________
Clinical diagnoses: ____
MRI diagnoses: ____
____
MR imaging technique and parameters: ____
____
Number of prints: ____
Diagnostic remarks: ____

Condylar morphology*

R		L
	No pathological findings/Convex	
	Gable-shaped/Pointed angle	
	Flattening	
	Concavity	
	Hypoplasia	
	Deformation	
	Fracture	
	**Compacta**: No pathological findings	
	Erosion	
	Osteophyte formation	
	**Spongiosa**: No pathological findings	
	Degeneration	
	Inflammation/Tumor	

Fossa/tubercular morphology

R		L
	No pathological findings	
	Erosion	
	Osteophyte formation	
	**Angle of articular eminence**: average	
	pointed	
	obtuse	
	**Tubercle**: No pathological findings	
	Pointed configuration	
	Flattening	

*** Multiple answers permitted**

Disk morphology* (closed mouth)

R		L
	No evaluation possible	
	Biconcave	
	Biplanar	
	Overall thickening	
	Overall or central thinning	
	Thickening in the marginal area	
	Flattening in the marginal area	
	Deformation	
	Plicated	
	Spherical	
	Perforated	
	Destroyed/Fragmented	

Condyle/fossa relationship (closed mouth)

R		L
	Centric	
	Anterior orientation	
	Posterior orientation	
	Cranial orientation	
	Caudal orientation	
	Localizer image in the sagittal plane	
	Localizer image perp. to condylar long axis	
	Localizer image insufficiently aligned	
	Condyle not discernible in localizer image	

Condyle/fossa relationship (open mouth)

R		L
	Translation movement: Regular	
	Translation movement: Hypermobile	
	Translation movement: Reduced	
	Translation movement: No	
