# Supplementary material for: Evaluation of the TMJ by means of Clinical TMD Examination and MRI Diagnostics in Patients with Rheumatoid Arthritis
Source: Biomed Res Int. 2014 Aug 26;2014:328560. doi: 10.1155/2014/328560 (PMC4160621; doi:10.1155/2014/328560)
Supplement: Supplementary file 2 [file 328560.f2.pdf]

Disk position sagittal (closed mouth)

R		L
	No evaluation possible	
	Regular	
	Partial anterior disk displacement	
	Complete anterior disk displacement	
	Posterior disk displacement	
	Localizer image in the sagittal plane	
	Localizer image perp. to condylar long axis	
	Localizer image insufficiently aligned	
	Condyle not discernible in localizer image	

Disk position sagittal (open mouth)

R		L
	No evaluation possible	
	Regular	
	Anterior disk displacement	
	Posterior disk displacement	
	Disk reduction: Yes	
	Disk reduction: No	
	Disk adhesion	
	Localizer image in the sagittal plane	
	Localizer image perp. to condylar long axis	
	Localizer image insufficiently aligned	
	Condyle not discernible in localizer image	

Disk position coronal (closed mouth)

R		L
	No evaluation possible	
	Regular	
	Lateral displacement	
	Medial displacement	
	Anterior displacement	
	Localizer image parallel to condylar long axis	
	Localizer image insufficiently aligned	
	Condyle not discernible in localizer image	

Disk displacement right

	Anterior	Posterior	Regular
Regular			-----------
Lateral			
Medial			

Disk displacement left

	Anterior	Posterior	Regular
Regular			-----------
Lateral			
Medial			

Bilaminar zone (sagittal/open mouth)

R		L
	No evaluation possible	
	Discotemporal ligament visible	
	Discotemporal ligament not visible	
	Discocondylar ligament visible	
	Discocondylar ligament not visible	

Signal intensity, TSE T2-weighted (condyle)

R		L
	No increased signal intensity	
	Increased signal intensity	

Signal intensity, TSE T2-weighted (joint space/bilaminar zone)

R		L
	No increased signal intensity	
	Increased signal intensity	

MRI with contrast media, joint/soft tissue/bone:

Signature:
